# Mineral pitch induces apoptosis and inhibits proliferation via modulating reactive oxygen species in hepatic cancer cells

**DOI:** 10.1186/s12906-016-1131-z

**Published:** 2016-05-27

**Authors:** Kishor Pant, Parul Gupta, Preeti Damania, Ajay K. Yadav, Aanchal Gupta, Anam Ashraf, Senthil K. Venugopal

**Affiliations:** Faculty of Life Sciences and Biotechnology, South Asian University, Akbar Bhawan Chanakyapuri, New Delhi, India

**Keywords:** Mineral Pitch, Anticancer, Hepatocellular carcinoma, Oxidative stress, Proliferation

## Abstract

**Background:**

Mineral Pitch (MP) is a dark brown coloured humic matter originating from high altitude rocks. It is an Ayurvedic medicinal food, commonly used by the people of the Himalayan regions of Nepal and India for various body ailments.

**Methods:**

The Huh-7 cells were treated with different concentrations of MP for 24 h, and both apoptosis and proliferation was determined by the TUNEL and MTT assays respectively. The formation of ROS and nitric oxide was analysed by DCFH-DA and Griess reagent respectively. The expression of miRNA-21 and miRNA-22 were checked by the real time PCR. Effect of miRNA-22 on proliferation and c-myc was studied by over-expressing miRNA-22 premiRs in Huh-7 cells.

**Results:**

We found that MP enhanced anti-cancer effects by inducing apoptosis and inhibiting proliferation. MP induced both ROS and NO, upon neutralizing them, there was a partial recovery of apoptosis and proliferation. MP also induced miRNA-22 expression, while miRNA-21 expression was inhibited. Over-expression of miRNA-22 resulted in a significant inhibition of proliferation. miRNA-22 directly targeted c-myc gene, thereby inhibited proliferation. These results clearly show that MP induces its anti-cancer activity by more than one pathway.

**Conclusion:**

The data clearly indicate that MP induced apoptosis via the production of ROS, and inhibited proliferation by inducing miRNA-22 and inhibiting miRNA-21 in Huh-7 cells.

## Background

Hepatocellular Carcinoma (HCC) is a complex form of neoplasm, associated with many risk factors such as, Hepatitis B and C virus infection, non-alcoholic fatty liver disease (NAFLD), alcohol abuse, aflatoxins, diabetes, obesity, and genetic factors [1]. HCC is the third leading cause of death among the cancer-related problems, and the prime cause of mortality among the cirrhosis patients [2].

Surgical resection is the best therapeutic preference for non-malignant primary liver tumour. The number of patients who undergo liver transplants are very less, which is due to the long waiting list, less number of transplant surgeons, donor availability problems and high cost. Several chemotherapeutic drugs have been assayed for the treatment of HCC [1]. However, success has been achieved only in few patients, or some with inequitable response. The major scientific challenge against HCC is the limited response to the available chemotherapy and the development of resistance during the treatment. Resistance to chemotherapy may be due to an improved DNA repair capacity and greatly activated antioxidant enzymes in the cancer cells. HCC is considered highly resistant to the therapeutic agents, leading to the DNA damage [1].

Approximately, 50  % of the modern anticancer drugs used in the cancer chemotherapy have been originated from the natural products. Hence, the use of natural products in the development of new drugs has been a great interest for researchers.

Recently several work has been done with Traditional Chinese Medicine (TCM) for the treatment of cancer. TCM has been used as adjuvant therapy to inhibit cancer, when Western medicines cannot provide any treatment options. TCM is used in conjunction with chemotherapy and radiotherapy for inhibiting the toxic effects of the treatments, as well as improving overall efficacy [3, 4].

Mineral Pitch (MP), also called as shilajit in local vernacular, is a dark brown coloured humic matter that drips out of the high altitude rocks (above 1000 m) during summer months. It is believed that it can cure almost all body ailments [5–7]. MP is a natural medicinal food, mainly used to treat people with weakness, inflammation, bone fracture, bleeding and for wound healing [6]. Since there is an insufficient number of medical facilities, eighty percent of total population mostly rely on the natural products for their primary health care needs [8].

MP is a humic matter, shown to contain fulvic acid and humic acid, which are responsible for its biochemical activities. In some previous studies humic matter has been reported to be anticancer agent as it inhibited the cancer cell growth and induced the apoptosis [9]. Moreover the cytotoxic properties of humic acid was accompanied the ROS production [10] and NO synthesis [11]. MP has been reported to be useful in reducing inflammation, arthritis, rheumatism, pain, ulcer, anxiety, stress, and diabetes [6]. Recently, MP also has been reported as an antiviral agent against Herpes Simplex Virus (HSV) [12] however, many of its other potential functions including its effects on cancer, has not been studied so far.

In this study, the anticancer property of MP was determined in hepatic cancer cells. It is an essential ethno-medicinal food, has large demand due to many therapeutic benefits in remedial recipes. Since a large population of Nepal and India customarily consume MP, the present investigation might have some implication in understanding its therapeutic significance in HCC management.

## Methods

### Collection of Mineral Pitch (MP)

MP was collected from the village Matela, Baitadi district of far-western Nepal, from the local inhabitants. This drug was transported to the laboratory in a clean and sterile bag. The stock solution was made by dissolving 2 mg/ml in double distilled water and was stored after filtering with 0.44 μm membrane filter and used for all the experiments.

### Cell culture and MP treatment

Huh-7 cells (1 × 10^6^) were cultured in 6-well plates using Dulbecco's modified Eagle's medium (DMEM) (Life Technologies, Carlsbad, USA) supplemented with 10 % fetal bovine serum (FBS) and 1 % penicillin/streptomycin. After 24 h, the cells were cultured in serum-free media and further incubated with different concentrations (0, 10, 20, 50, 100, 500, and 1000 μg/ml) of MP for 24 h. After incubation, the cells were washed and collected either for various assays, RNA isolation for RT-PCR or Western blots.

### ROS and NO neutralization experiments

Huh-7 cells were cultured in 6-well plates and the medium was replaced with serum-free media and the cells were incubated with MP (0, 50 and 100 μg/ml) for a period of 24 h. For ROS and NO neutralizing experiments, once the cells were replenished with serum-free media, the cells were pre-incuated with either N-acetyl cysteine (NAC, 5 mM) or L-N^G^-Nitroarginine methyl ester (L-NAME, 10 μM) for 1 h followed by the addition of MP (100 μg/ml) for a further period of 24 h. After the incubation, the cells were washed with PBS and were dissociated using PBS-2 mM EDTA and then stained with Annexin V or PI. Immediately the positive cells were counted using flow cytometry and the apoptosis induction was calculated.

### Cell viability assay

Huh-7 cells (5 × 10^3^ cells per well) were plated in 96-well plates. After 24 h the media was replaced with serum-free media along with various concentrations of MP for a further period of 24 h. MTT reagent (10 μl; 5 mg/ml) solution was added to each well and the plates were incubated at 37 °C for 1 h. The medium was removed, 100 μl of DMSO was added to each well and mixed for 5 min. The absorbance was measured at 570 nm and the untreated control cells were considered as 100 % of cell survival. The following formula was applied to calculate the cell viability [13].

$$ \mathrm{Cell}\;\mathrm{viability}\;\left(\%\right)=\frac{\mathrm{Absorbance}\;\mathrm{of}\;\mathrm{control}-\mathrm{Absorbance}\;\mathrm{of}\;\mathrm{M}\mathrm{P}\;\mathrm{treated}\;\mathrm{Well}}{\mathrm{Absorbance}\;\mathrm{of}\;\mathrm{control}}\times 100 $$

### Colony formation assay

The 2.0 × 10^3^ cells were seeded in 60 mm cell culture-treated dishes, along with the various concentrations of the MP (10–1000 μg/ml). The cells were incubated for 6 days, with a change of media and MP at every 24 h. At the end of 6 days, 0.5 % crystal violet dissolved in ethanol was used to stain the cells and the data were calculated [14].

### TUNEL assay

The cells were cultured in 6-well plates with various treatments. After the incubation, TUNEL assay was performed using in situ cell death detection kit (Roche Diagnostics, USA) as described by us previously [15]. The percentage of cell death was calculated by following formula:

$$ \left[\mathrm{Percent}\;\mathrm{of}\;\mathrm{apoptotic}\;\mathrm{cell}\mathrm{s}=\mathrm{TUNEL}\;\mathrm{positive}\;\mathrm{cell}\mathrm{s}/\mathrm{Total}\;\mathrm{cell}\;\mathrm{count}\times 100\right]. $$

### ROS determination

Huh-7 cells were cultured (5 × 10^5^ cells/well) for 24 h and the media was replaced with serum-free media along with different concentrations of MP for a further period of 24 h. A solution of 2’,7’-dihydrochloroflurorescein acetate (DCFH-DA) (10 μM) was added to the cells and incubated for 60 min in dark. After staining, the cells were collected using PBS containing 2 mM EDTA. The fluorescence of DCFH-DA labelled cells were examined using flow cytometry analysis.

### NO determination

The cells (5 × 10^5^ cells per well) were incubated with different concentration of MP for 24 h. After the incubation, the media was collected and used for the estimation of the production of NO using Nitrate/Nitrite Colorimetric Assay Kit (Cayman, USA) as per the manufacturer’s instructions. The percent of nitrite production was calculated against untreated control.

### Lipid peroxidation assay

After the MP treatment, the cells (5 × 105 cells per well) were lysed. The homogenate (1 ml) was mixed with 0.15 M Tris–Cl buffer (pH 7.4), 10 % trichloroacetic (TCA) and 50 mM thiobarbituric acid (TBA). This mixture was heated for 30 min at 80 °C, cooled and centrifuged for 10 min at 3000 rpm. The absorbance of the supernatant was measured against the blank (distilled water) at 530 nm in a UV spectrophotometer and MDA formed in the cells was determined.

### Assay for cellular antioxidant enzymes

The cells were incubated with different concentration of MP. After the incubation, reduced glutathione (GSH), catalase and SOD activity assays were performed according to the methods described previously [16, 17]. The total cellular protein content was estimated using BCA reagent (Thermo Scientific, USA) as per the manufacturer’s instructions. The protein content was used for the calculation of the enzyme activities.

### RNA isolation, cDNA synthesis, and Real-time PCR

Total RNA enriched with miRNA was extracted from Huh-7 cells using the mirVana miRNA isolation kit (Life Technologies, Carlsbad, CA, USA), following the manufacturer’s instructions. Reverse transcription was performed using the Universal cDNA synthesis kit (Exiqon, Vedbaek, Denmark). RT-PCR was done with SYBR Green and PCR master mix (Life Technologies). Cyber-green method was used for the real-time PCR using specific primers for both miRNA-21 and miRNA-22, and 5S RNA was used as an internal control as described previously [18, 19]. Each PCR was performed in duplicates and the data were normalized with endogenous 5S RNA levels. Relative expression were calculated using 2^-ΔΔct^ values. All the experiments were repeated at least three times in triplicates.

### MiRNA-22 transfection experiments

Huh-7 cells cells (3.5 × 10^5^ cells/well) were plated in 6-well plates and transfected with either miRNA-22 (10nM) or a non-specific miRNA (10nM; a miRNA which does not inhibit any known mRNA) (Sigma Aldrich, USA) using siPORT miRNA transfection reagent (Invitrogen, USA) as per the manufacturer’s instructions. In parallel, the cells were transfected with fluorescein conjugated siRNA to check the transfection efficiency. After 24 h, the media was changed and allowed to grow for 48 h and then the cells were collected either for RNA isolation or protein isolation.

### Protein isolation and Western blot analysis

After 72 h of transfection, the total cellular protein was isolated from the transfected cells using mammalian protein extraction buffer (Thermo Scientific, USA). Protein concentration was estimated using bicinconinic acid (BCA) protein estimation kit (Thermo Scientific). Samples (60 μg/lane) were run on a 12 % SDS-PAGE gels, and transferred to polyvinylidene fluoride membranes. Western blots were analyzed using antibodies for c-myc protein and β-actin (Cell signalling, USA). After washing and developing using ECL kit (Thermo Scientific), the protein bands were visualized.

### Statistical analysis

All the experiments were conducted in duplicates and at least three independent experiments. The data were calculated and expressed as mean ± standard deviation (SD). The analysis of variances (ANOVA) was calculated among the groups followed by the Student’s t-test for the differences between the groups. The level of significance was computed and the values were considered significant when *p* < 0.05.

## Results and discussion

MP contains 60–80 % humic matter (humic acid, fulvic acid and humin), and rest of the content may be secondary metabolites from the plant and/or animal origin [6]. The presence of several contents might be due to the plant/animal origin of MP or addition of various medicinal plants (*i.e.* Ashwagandha, Triphala or Tulsi) during the cooking process, which could augment the therapeutic properties of MP against various health complaints. In the present study, we demonstrated the anticancer properties of MP, by performing proliferation and apoptosis assays.

### MP inhibits cell proliferation

**Fig. 1:**
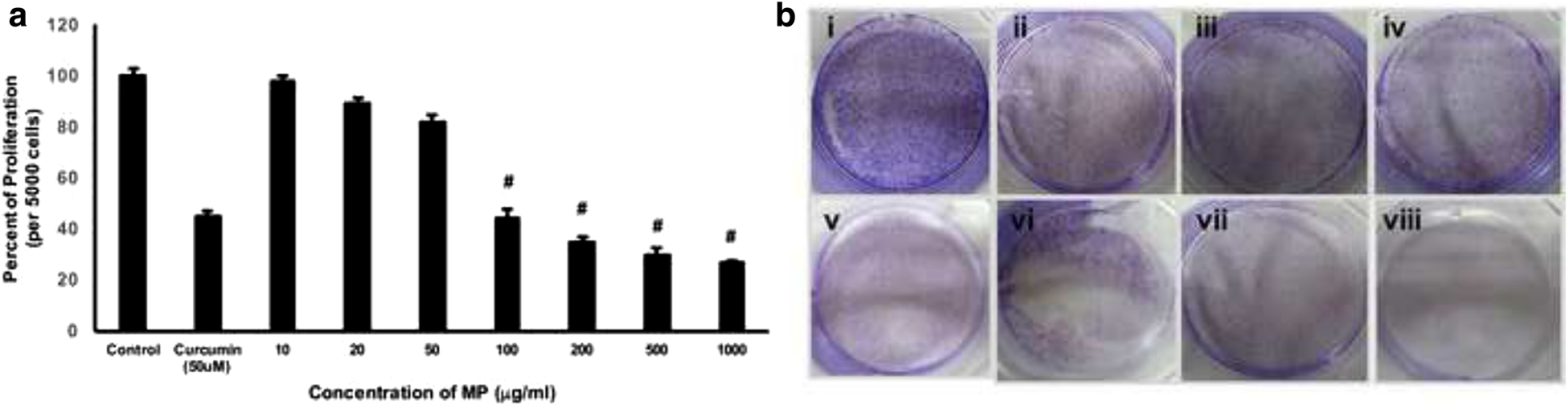
Huh-7 cells (5,000 cells per well of 96-well plates) were cultured with different concentrations of MP for 24 h under serum-free conditions. **a** MTT assay was performed and the percent of proliferation was calculated. In parallel experiments, curcumin (50 μM) was used as a positive control (*n* = 3; ^#^ *p* < 0.05). **b** The colony formation assay was performed after plating cells in 60 mm dishes and incubating them with different concentrations of MP for 6 days. After that the cells were stained with 0.5 % crystal violet and the colonies were visually observed. The representative pictures show, untreated cells (i); cells treated with different concentrations of MP, 10 μg/ml (ii), 20 μg/ml (iii), 50 μg/ml (iv), 100 μg/ml (v), 200 μg/ml (vi), 500 μg/ml (vii), and 1000 μg/ml (viii)

First, the effect of MP on cell proliferation and survival was estimated. The cell proliferation assay was performed using MTT assay. It is a NADPH-dependent cellular oxidoreductase enzyme assay and produces insoluble purple colour formazan, which represents the numbers of the viable cells [20]. The insoluble crystals are dissolved using DMSO and then quantitated. Upon incubation of the cancer cells with MP, the proliferation was significantly decreased and was directly proportional to the increasing concentration of MP. There was a 55.8, 65.3, 70.3 and 73.3 % (*p* < 0.01) reduction in the cell proliferation when the cells were incubated with 100, 200, 500 and 1000 μg/ml concentration of MP respectively (Fig. 1a). The lower concentrations of MP did not have any significant effects. Next, colony formation assay was performed and found that the inhibition of cell number was well correlated with the MTT assay (Fig. 1b). The inhibition in the colony formation was indicated by the observation of the blue coloured colonies, and the maximum inhibition was observed in the 1000 μg/ml (Fig. 1b, viii), followed by 500 μg/ml (Fig. 1b, vii), 200 μg/ml (Fig. 1b, vi) and 100 μg/ml (Fig. 1b, v) concentrations. Both MTT assay and colony formation assay showed the inhibition of Huh-7 cells by MP treatment, which indicates its anti-proliferative properties in tumour cells.

### MP induces apoptosis

**Fig. 2:**
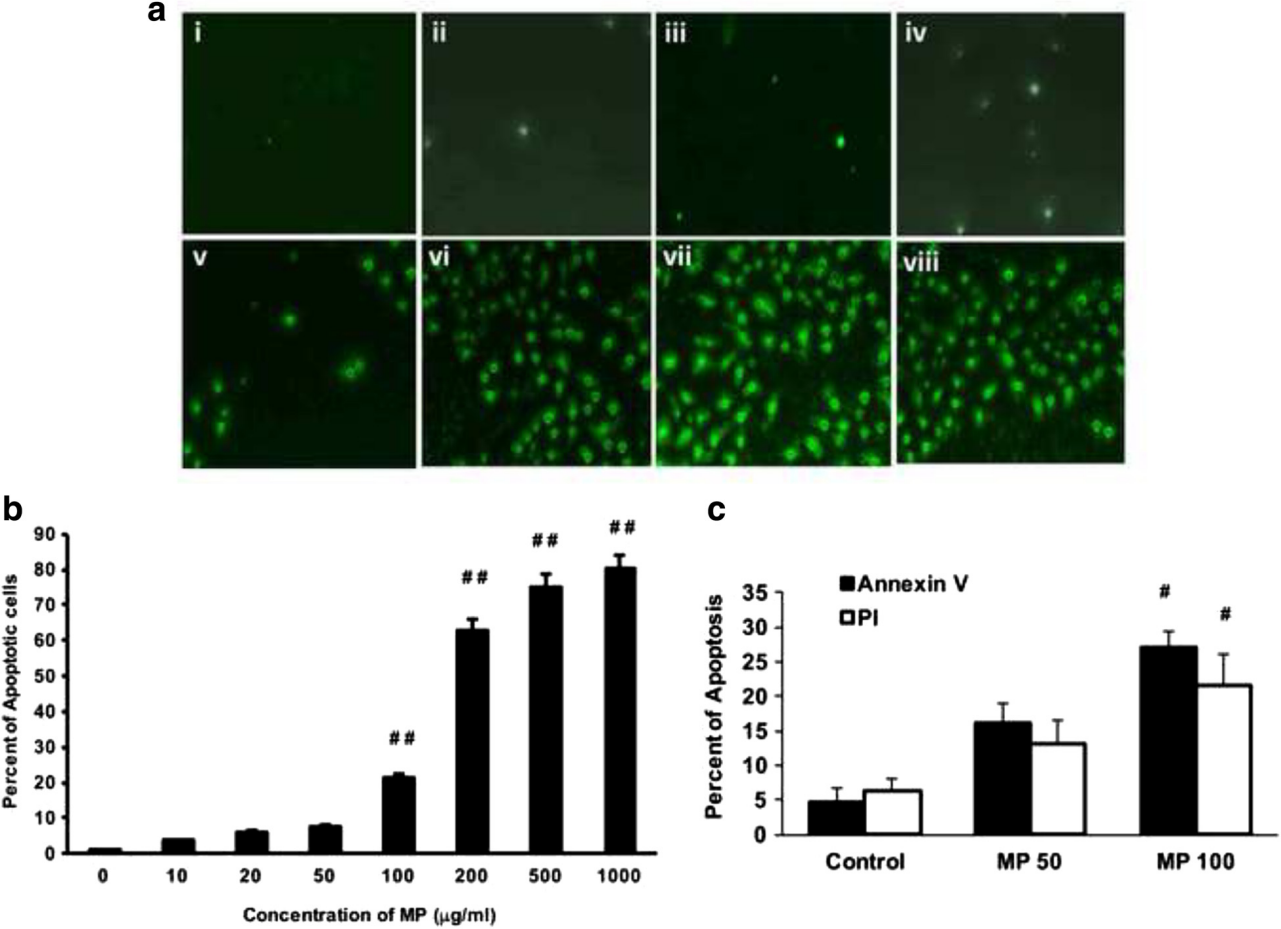
Huh-7 cells were cultured in 6-well plates and incubated with different concentrations of MP. **a** The cells were incubated with different concentration of MP for 24 h and TUNEL assay was performed. The pictures are representative from three different experiments. (i) untreated control cells, and (ii) 10 μg/ml, (iii) 20 μg/ml, (iv) 50 μg/ml, (v) 100 μg/ml, (vi) 200 μg/ml, (vii) 500 μg/ml and (viii) 1000 μg/ml of MP. **b** The positive cells were counted in at least 10 different high power fields in three different experiments and the average was plotted as percent of control (*n* = 3; ^##^ *p* < 0.01). **c** To confirm the induction of apoptosis, cells were incubated with two different concentrations of MP (0, 50 and 100 μg/ml). The cells were collected and Annexin V and PI exclusion assays were performed using Flow cytometry and the percent of apoptosis was calculated (*n* = 3; ^#^ *p* < 0.05)

Next, the effect of MP on inducing apoptosis was evaluated as described in the Methods section. TUNEL assay was performed to detect the for DNA fragmentation detection, which could be a result of apoptosis [21]. The apoptosis induction was increased with increasing concentrations of MP (Fig. 2a). The apoptotic and the total cells were counted in at least 10 different high power fields and the average of apoptotic cells were calculated. The control cells were used as 1and the percent of the apoptotic cells were found to be 21.7, 63, 77.3 and 80.1 % with the concentrations 100, 200, 500 and 1000 μg/ml of MP compared to the control untreated cells (*p* < 0.01). While, the treatment of other concentrations did not have any significant increase in the apoptosis (Fig. 2a and b). The apoptosis induction was confirmed by Annexin V and PI exclusion assays. Two different concentrations of MP (50 and 100 μg/ml) were selected and the apoptosis induction was measured by flow cytometry. Although there was a increase with MP (50 μg/ml), significant increase was observed with 100 μg/ml of MP (Fig. 2c). These results confirm the TUNEL assay findings.

Resistance to chemotherapy in tumour cells is due to enhanced DNA repair capacity. HCC is considered to be a tumour which is highly resistant to agents attacking DNA. Several herbal composite formulas and natural components (Curcumin, Resveratrol, and Silibinin) have been shown to be beneficial for the cancer chemoprevention via inducing the DNA damage [22]. In the present study, for the first time we have reported the anti-proliferative and pro-apoptotic properties of the MP in hepatic cancer cells.

### MP induces both ROS generation and NO production

**Fig. 3:**
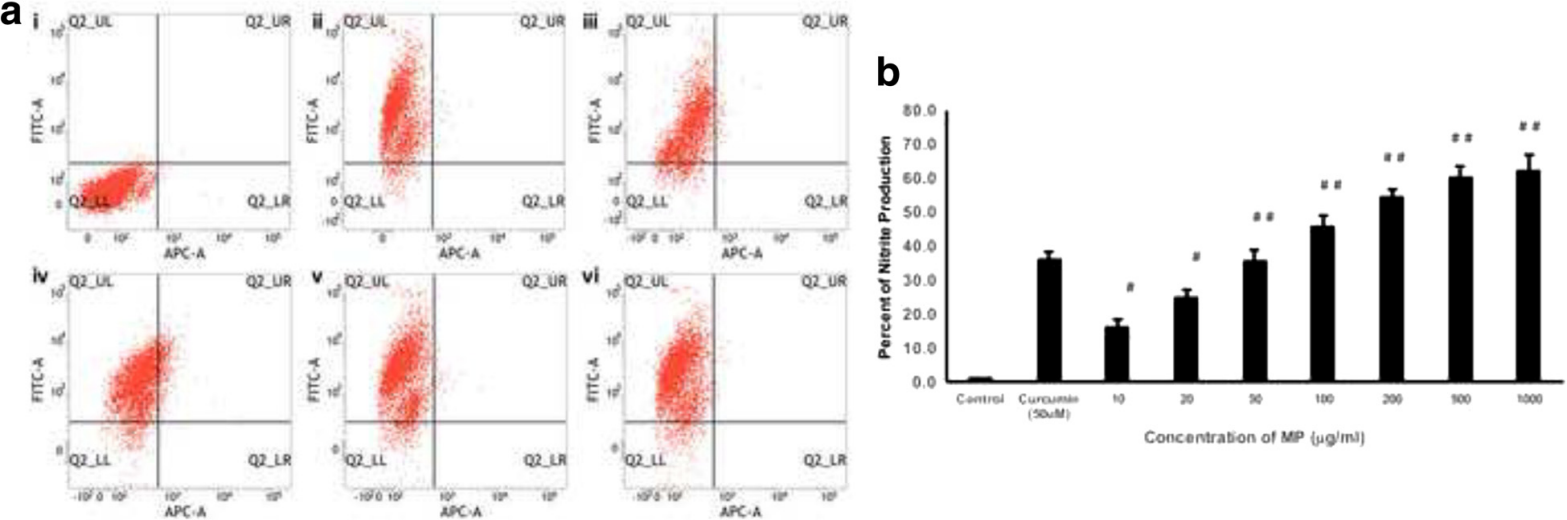
Huh-7 cells were cultured in 6-well plates with different concentrations of MP. **a** The cells were stained with 10 μM DCF-DA and kept in dark for 60 min. The cells were resuspended in PBS-EDTA solution and flow cytometry was performed to determine the ROS release. (i) untreated control cells, and (ii) 10 μg/ml, (iii) 20 μg/ml, (iv) 50 μg/ml, (v) 100 μg/ml, (vi) 200 μg/ml and (vii) 500 μg/ml. **b** Nitric oxide release was measured in terms of nitrite production using Griess reagent. After the incubation with different concentrations of MP, Griess reagent was added to the medium and the nitrite formation was measured using Nitrate/nitrite colorimetric assay kit (Cayman). In parallel experiments, curcumin (50 μM) was used as a positive control (*n* = 3; ^#^ *p* < 0.05 and ^##^ *p* < 0.01)

The cells were incubated with different concentrations of MP and the intracellular ROS levels and the nitrite levels in the media were measured. There was a 30, 38 and 49.8 % increase in ROS production in the cells treated with 100, 200, and 500 μg/ml of MP treatment respectively (Fig. 3a). Evidence supports that oxidative stress-induced apoptosis may play an important role in the anti-carcinogenic effect of several chemopreventive agents (e.g. retinoids, nonsteroidal anti-inflammatory drugs, polyphenols, tamoxifen, vanilloids, and rotenoids) [23].

The Griess reagent is used to measure the level of NO produced in the cells. Since NO is highly unstable and gets converted to nitrite, the level of the nitrite was measured with increasing concentrations of the MP. The percentage of the NO production was 65.33, 61.2, 60.27, 51.26, 36 and 24.7 for 1000, 500, 200, 100, 50, and 20 μg/ml, reported respectively and these values were statistically significant (*p* < 0.01) (Fig. 3b). NO may lead to the cell death via inducing pro-apoptotic signals. During the lack of respiration, mitochondrial membrane potential is reduced, cytochrome c is released, the transition pores are opened and calcium is released by the increased NO level which eventually leads to apoptosis [21].

### Effect of MP on cellular antioxidants

**Fig. 4:**
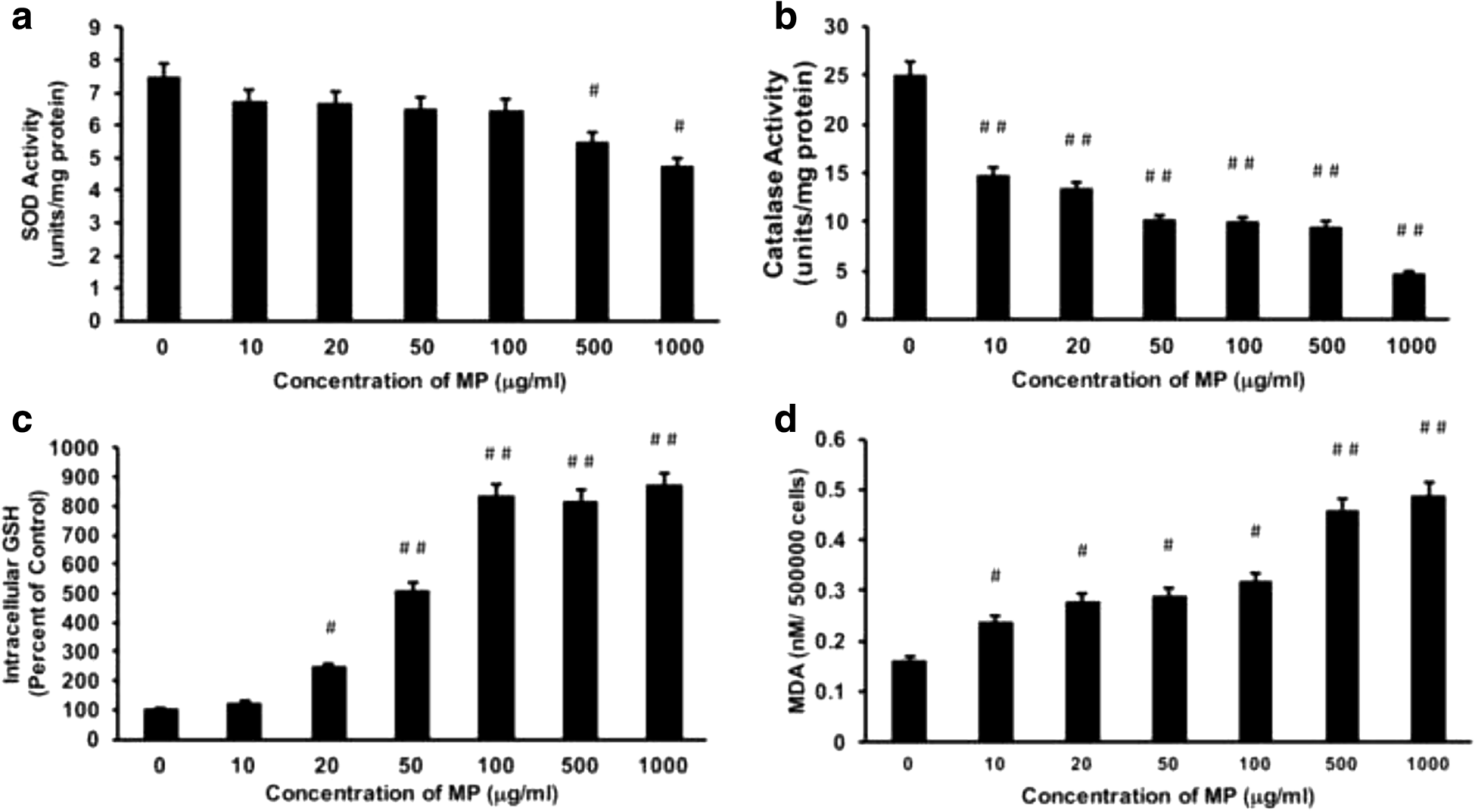
Huh-7 cells were cultured with different concentrations of MP for 24 h under serum-free conditions. After 24 h of treatment, SOD activity (**a**), catalase activity (**b**), intracellular GSH levels (**c**), and intracellular MDA levels (as a marker of lipid peroxidation) (**d**) were measured as described in Methods. The total cellular protein content was also measured and the results of the enzyme activity were expressed per mg protein (*n* = 3; ^#^ *p* < 0.05 and ^##^ *p* < 0.01)

To analyse the anti-oxidant levels in the cells, the cells were cultured with different concentrations of MP and the anti-oxidant levels were measured. The SOD activity in Huh-7 cells was found to be decreasing with increasing concentrations of MP. The SOD activity was found to be significantly decreased by 20 and 34 % (*p* < 0.05) only with high concentrations, 500 μg/ml and 1000 μg/ml, of MP respectively (Fig. 4a). The SOD activity was not changed with other concentrations of the MP. Our data showed that there was an increase in ROS production in the cells due to the MP treatment. Hence, the catalase activity was determined in these cells. The catalase activity was significantly decreased in all the concentrations of MP that was tested. There was a significant reduction of 40, 38, 45, 51, 53 and 75 % of catalase activity with the increasing concentrations of MP, 10, 20, 50, 100, 500, and 1000 μg/ml, respectively (Fig. 4b). These results suggest that there is an increased load of ROS levels in the cells incubated with MP. Next, the glutathione production was measured in the MP-treated cells. Glutathione production was significantly increased with the increasing concentrations of MP. There was a more than 7-fold increase of glutathione production with 100, 500 and 1000 μg/ml of MP (Fig. 4c).

Superoxide dismutase (SOD) converts superoxide into H_2_O_2_ and O_2_. The catalase (CAT) enzyme catalyses the decomposition of H_2_O_2_ to H_2_O and O_2_ [24]. These enzymes ensure that the cells effectively deal with ROS and free radicals, induced oxidative stress. We demonstrated that although MP simultaneously down-regulated SOD, CAT and while GSH was up-regulated in cancer cells, the cell death was increased. The falling level of the cellular antioxidants may be a reason for the augmented level of ROS in the cells.

The lipid peroxidation of the cancer cells was found to be increased in concentration dependent manner. There was a 2-fold increase with 50 and 100 μg/ml concentration of MP and there was a 3.3 fold increase in MDA levels with 500 and 1000 μg/ml concentration of MP respectively (Fig. 4d). It is the oxidative degradation of lipids by free radicals and ROS which capture electrons from the lipids of cell membranes, leading to the cell damage and finally results in the apoptosis [23]. In this study, we have clearly shown the *in-vitro* anti-cancer properties of MP through generation of the ROS and NO, which was accompanied by the lipid peroxidation-induced apoptosis in hepatic cancer cells.

### MP-induced ROS plays a role in proliferation and apoptosis

**Fig. 5:**
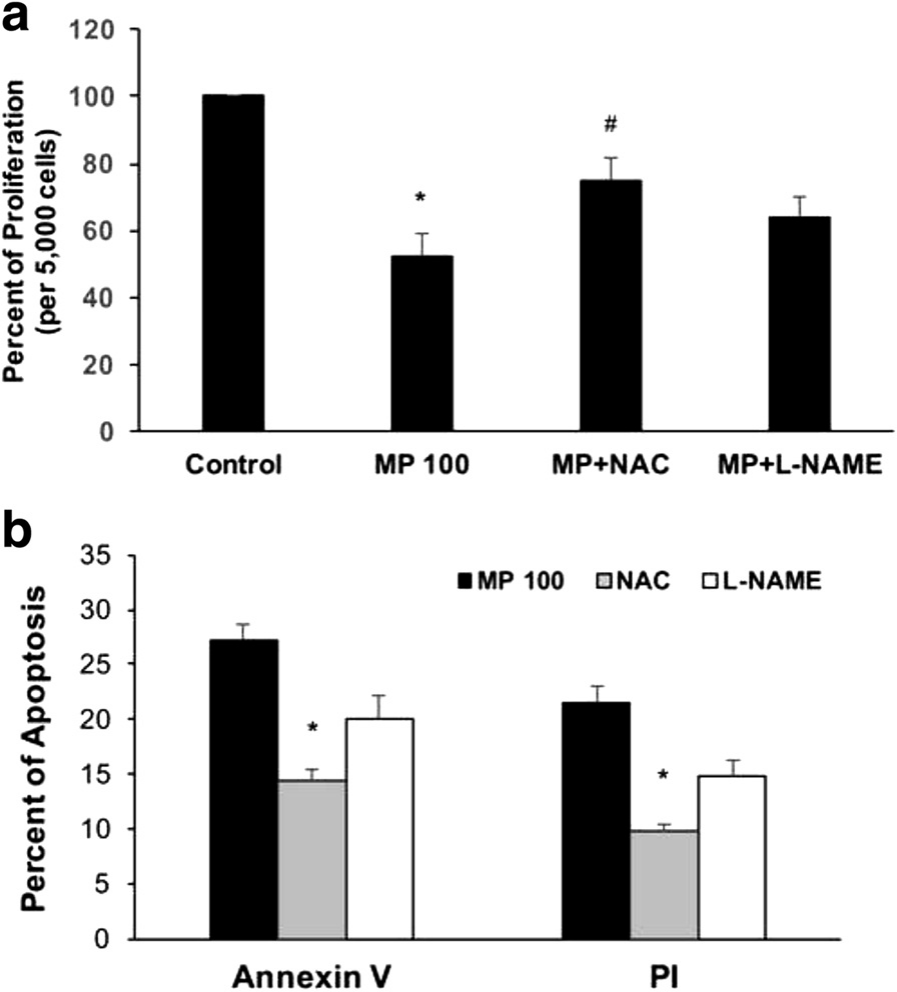
Huh-7 cells were cultured with MP (0 and 100 μg/ml) along with either NAC or L-NAME. **a** After 24 h of incubation, the cell proliferation was estimated using MTT as described in Methods (*n* = 3; *p < 0.05 compared to control; ^#^ *p* < 0.05 compared to MP 100 μg/ml). **b** The cells were incubated with MP (100 μg/ml) along with NAC or L-NAME. Apoptosis induction was determined using Annexin V and PI exclusion assays All the experiments were performed in triplicates and repeated at least three times (*n* = 3; *p < 0.05)

To confirm whether ROS and NO play a role in proliferation and apoptosis, the cells were incubated with MP (100 μg/ml) alone, or with NAC and L-NAME for 24 h. MTT assay was performed and found that there is a significant inhibition in proliferation (Fig. 5a). When the cells were incubated with MP and NAC, there was a partial recovery of the proliferation suggesting that ROS might play a significant role in partly inhibiting the MP-induced proliferation. When cells were incubated with MP with L-NAME, although there was a slight increase in proliferation, but was not significant. These data suggest that nitric oxide alone might not be sufficient to be responsible for the decreased proliferation, but may contribute indirectly along with ROS in cells. Next, the role of neutralizing ROS and NO on apoptosis was evaluated. The cells were incubated with MP (100 μg/ml) alone, or with NAC and L-NAME for 24 h. The cells were collected and flow cytometry was used to analyse the Annexin V and PI staining. There was a significant levels of apoptosis was found in MP alone treated cells. MP-induced apoptosis was recovered, at least in part, by the addition of NAC, but addition of L-NAME did not result in a significant recovery of apoptosis (Fig. 5b), suggesting that NO alone might not have a significant role in MP-induced apoptosis. It might exert its effect along with ROS in these cells.

### MP decreases the expression of miRNA-21 and increases the expression of miRNA-22

**Fig. 6:**
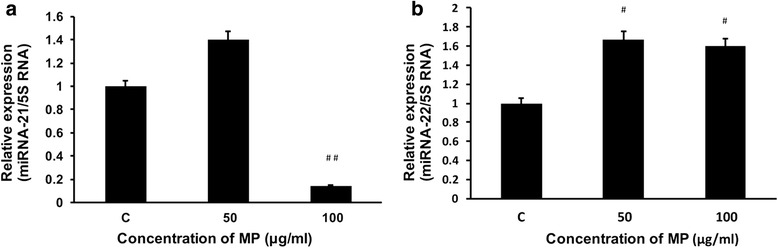
Huh-7 cells were culture in 6-well plates with different concentrations of MP (0, 50 and 100 μg/ml). After 24 h of incubation, the cells were collected, total RNA enriched with miRNAs were isolated, cDNA was synthesized and real time RT-PCR for miRNA-21 (**a**) and miRNA-22 (**b**) was performed as described in Methods. 5S RNA was used as internal control in all these real time PCR experiments. All the experiments were performed in triplicates and repeated at least three times (*n* = 3; ^#^ *p* < 0.05 and ^##^ *p* < 0.01)

Expression of miRNA-21 was found to be decreased (85.5 % of control) significantly with the 100 μg/ml of the MP as compared to the untreated control cells (Fig. 6a). 50 μg/ml MP treated cells showed a 1.67 fold increase and 100 μg/ml of MP treated cells showed the 1.6 fold increase in the expression of miRNA-22 (Fig. 6b).

miRNA-21 is a specific oncomiR (cancer specific MiRNA), found more abundant in human cancers like lung, pancreas, skin, liver, gastric, cervical, thyroid, and various lymphatic and hematopoietic cancers. Inhibition of miRNA-21 in HCC lines reduced the phenotypic behaviours of cancer cells (e.g. decreased cell proliferation, migration and invasion). It contributes to the enhanced aggressiveness of HCC and consequently results in poor diagnosis in HCC patients. It induces the proliferation of cancer cells by the repressing the PTEN (a tumour suppressor gene) [25]. Previously we have shown that over-expression of miRAN-21 increases the proliferation in heaptic cancer cells [18]. The data suggest that MP might inhibit proliferation, at least in part, via inhibiting miRNA-21 in these cells.

miRNA-22 has been observed as a tumour suppressing agent in many cancers including HCC [26]. A known target of miRNA-22 is histone deacetylase 4, which is known to have many important functions in cancer development and proliferation [26]. MP treatment lead to the reduction of the miRNA-21 while it increased the level of the miRNA-22 expression significantly, which indicates the anti proliferative activities of MP *via* regulating the epigenetic factors.

### Over-expression of miRNA-22 decreases proliferation in Huh-7 cells

**Fig. 7:**
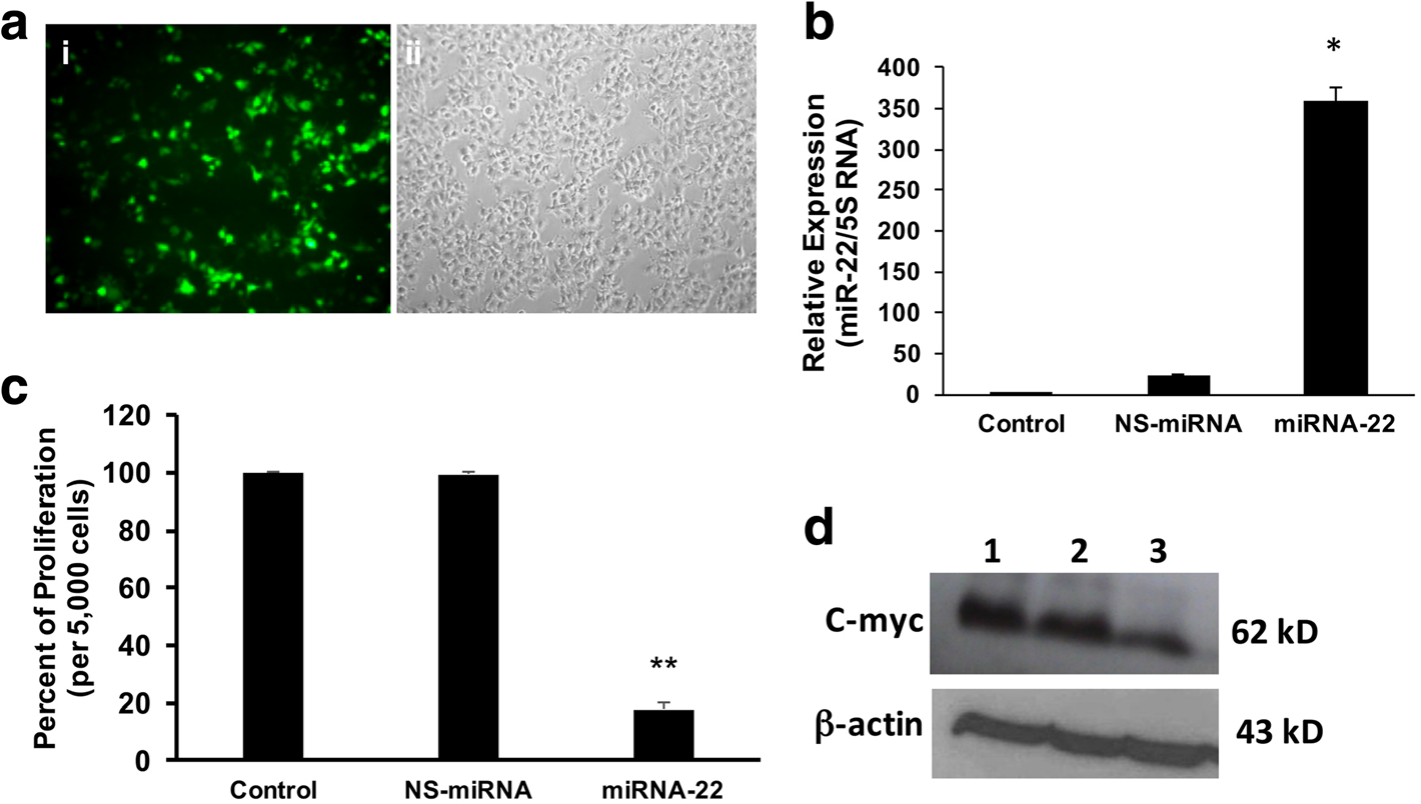
Huh-7 cells were culture in 6-well plates and transfected with either fluorescein-conjugated siRNA, non-specific miRNA (NS-miRNA) or miRNA-22 for 72 h as described in Methods. **a** The cells were observed under the fluorescent microscope (i) and the same field was observed under phase-contrast microscope (ii). **b** Total RNA was isolated, cDNA was synthesized and real time RT-PCR was performed for miRNA-22 and 5S RNA. The relative expression was calculated and presented (*n* = 3; *p* < 0.001). **c** MiRNA-22 over-expressing cells were analyzed for the proliferation assay using MTT (*n* = 3; *p* < 0.01). **d** The total cellular protein was isolated from miRNA-22 over expressing cells and Western blot was performed for c-myc and b-actin protein. The picture is a representative of three experiments. Lane 1, control; lane 2, NS-miRNA; lane 3, miRNA-22

MP treatment caused increased levels of miRNA-22. To test whether over-expression results in decreased proliferation, we incubated cells with non-specific miRNA, which is shown not to inhibit any known mRNA, or miRNA-22 preimiRs for 72 h. In parallel experiments, to check the efficiency of the transfection, the cells were transfected with fluorescein conjugated siRNA under similar conditions. The results showed more than 75 % of the cells were transfected with the siRNA (Fig. 7ai) and corresponding phase-contrast image has been shown (Fig. 7a, ii). To check the intracellular levels of miRNA-22 after the transfection experiments, the cells were collected, total RNA enriched with miRNAs were isolated, cDNA was synthesized and real time PCR was performed using miRNA-22 primers or 5S RNA, as control. The relative expression was calculated and the results show that there is a significant increase in miRNA-22 in the transfected cells (Fig. 7b). Next, its role on the proliferation was studied by MTT assay. The cells were plated with or without miRNA-22 or with NS-miRNA. The cell proliferation was estimated and found that there was a 4.9 fold decrease in miRNA-22 transfected cells (Fig. 7c). Next, we studied one of its target proteins, c-myc, expression in these cells. Hence, the transfected cells were isolated for the total cellular protein and Western blots were performed for c-myc and β-actin, as a loading control. The results showed that there was a 60 % reduction in the c-myc protein levels in the miRNA-22 transfected cells (Fig. 7d). These results confirm that MP-induced miRNA-22 is partly responsible for decreased proliferation in hepatic cells. Recently it has been shown that miRNA-22 inhibits tumor growth and metastasis in gastric cancer [27]. Another study also has shown that miRNA-22 is a tumor suppressor [28] confirming that our results are well correlated with others findings. Hence, the data from this study suggests that MP inhibits proliferation more than one pathways in hepatic cancer cells. MP induces ROS and nitric oxide, enhances the expression of miRNA-22 and decreases the expression of miRNA-21, a known onco-miR. The enhanced apoptosis could be due to the increased ROS, although nitric oxide might not play alone in inducing apoptosis, along with increased ROS, it might have an added effect to induce apoptosis in hepatic cancer cells.

Throughout the centuries of herbal and natural medicinal practice, there are a number of candidate drugs derived from the herbs or herbal compounds for chemotherapeutic approach against HCC. Certain herbal compounds have been found as an anti-HCC agent and are successfully in use for cancer therapy. In this manuscript we have shown that MP could be developed as a potential natural chemotherapeutic agent.

## Conclusion

**Fig. 8:**
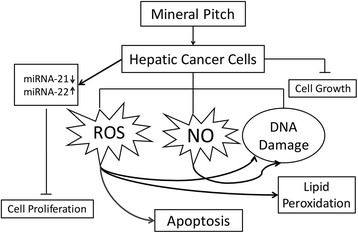
Illustration shows the overall mechanism by which MP could exert its anticancer activity in the hepatic cancer cells

MP vastly used in western Nepal and Kumaun (India), for a number of health complaints. In our study, MP had shown as potential anti-proliferative and pro-apoptotic properties *via* ROS and NO, as well as by modulating the expression levels of miRNA-21 and miRNA-22 (Fig. 8). The evidence from the present study suggests that MP may be a factor in diet that may lower the risk of cancer and may inhibit the tumour growth. MP has been consumed for centuries by the people for a variety of purposes. Our study for the first time provides an evidence that proliferation, apoptosis, oxidative stress and miRNAs are regulated by MP.

## Abbreviations

CAT, Catalase; DCFDA, 2’,7’ dichloro-fluorescein diacetate; DMSO, Dimethyl sulfoxide; L-NAME, L-N^G^-Nitroarginine methyl ester; MDA, malondialdehyde; MTT, 3-(4,5-dimethylthiazol-2-yl)-2,5-diphenyltetrazolium bromide; NAC, N-acetyl cysteine; NO, Nitric oxide; PBS, Phosphate buffer saline; ROS, Reactive oxygen species; RT-PCR, Real time PCR; SOD, Superoxide dismutase; TUNEL, Terminal deoxynucleotidyl transferase dUTP nick end labelling.
